# Plasma Kisspeptin Levels in Girls with Premature Thelarche

**DOI:** 10.4274/jcrpe.615

**Published:** 2012-06-09

**Authors:** Ayşehan Akıncı, Dilek Çetin, Nevin İlhan

**Affiliations:** 1 İnönü University, Turgut Özal Medical Center, Department of Paediatric Endocrinology, Malatya, Turkey; 2 Fırat University Faculty of Medical, Department of Biochemistry, Elazığ, Turkey; +90 422 341 06 60aakinci@inonu.edu.tr

**Keywords:** Kisspeptin, premature thelarche

## Abstract

**Objective:** Premature thelarche (PT) is defined as isolated breast development without secondary sex characteristics in girls below the age of eight. We aimed to determine whether the level of kisspeptin, which plays a role in the release of gonadotropins, is associated with PT.

**Methods:** The patient group included children with PT aged 3-8 years (n=20) and the control group included healthy children in the same age range (n=20). Height standard deviation scores (HSDSs), bone maturation and growth velocity were evaluated in the two groups. Basal follicle-stimulating hormone (FSH), luteinizing hormone (LH), estradiol (E2), prolactin (PRL), and sex hormone-binding globulin (SHBG) levels were also measured in the two groups by immunochemiluminometric assay (ICMA). A gonadotropin-releasing hormone (GnRH) test was also conducted in the patient group and the peak levels of FSH and LH were determined. Kisspeptin levels were measured using enzyme immunoassay (EIA).

**Results:** No differences were found between the groups in terms of age, HSDS, annual growth rate and bone age. While the plasma basal FSH, LH and E2 levels in the patient and control groups did not show statistically significant differences, PRL levels were higher in the patient group (p<0.05). Peak LH response to GnRH test was at the prepubertal level (<5 ng/mL) in patients with PT. In the patient group, kisspeptin levels were significantly higher compared to the levels in the control group (2.96±1.21 ng/dL vs. 1.19±0.41 ng/dL; p<0.05), and kisspeptin levels showed a significant correlation with PRL, FSH, LH, and E2 levels (p<0.05).

**Conclusions:** In this study, plasma kisspeptin levels were found to be higher in patients with PT and to show a positive correlation with increased PRL levels. Kisspeptin is one of the neuropeptides that plays a role in the onset of puberty. Our results support the hypothesis that PT may result from the temporary activation of central stimulants.

**Conflict of interest:**None declared.

## INTRODUCTION

Breast enlargement or thelarche is the first sign that indicates the onset of physiological puberty in girls. On the other hand, premature thelarche (PT) is defined as isolated breast development without other features of puberty (e.g. accelerated growth velocity, advanced bone maturation, axillary and pubic hair development) in girls below the age of 8 years. Although the onset of PT shows a peak around the first two years of life, it may also occur during any period between the ages of 2 and 8 years (1,2). PT may be a mild benign form of hypothalamic-pituitary-gonadal (HPG) axis activation and occasionally, may develop as a result of an accelerated maturation of HPG axis activation. It may show rapid progression to precocious puberty, with an incidence of approximately 14% (3). The pathophysiology of PT remains to be elucidated. Various mechanisms are responsible for PT, such as increased sensitivity of breast tissue to estrogen (4,5), temporary estrogen release from ovarian follicular cysts (4), increased estrogen production from adrenal precursors such as dehydroepiandrosterone sulphate (6), exposure to exogenous estrogens (7), increased aromatase enzyme activity (8), relative increase of estrogen related to increased level of sex hormone-binding globulin (SHBG) (9), and temporary activation of HPG axis (10). The main hormones that contribute to breast development in puberty include estradiol (E2), progesterone, prolactin (PRL), growth hormone, and several growth factors such as insulin-like growth factor-1 and epidermal growth factor (11). The mechanisms activating these hormones in PT, which act without inducing other signs of puberty, has not been fully understood. Kisspeptin is a neuropeptide, encoded by Kiss-1 gene. Kisspeptin and the G protein-coupled receptor 54 system (GPR54/Kiss1R) are recognized as essential regulators of puberty onset and of gonadotropin secretion (12) . It has been found that the kisspeptin/GPR54 complex stimulates HPG axis activation and regulates energy metabolism (13,14,15). Recent studies have shown that kisspeptin levels are significantly higher in girls with central precocious puberty than in prepubertal girls, and this finding is positively correlated with peak levels of follicle-stimulating hormone (FSH) and luteinizing hormone (LH) after gonadotropin-releasing hormone (GnRH) stimulation (16,17). For these reasons, it is speculated that kisspeptin plays an important contributive role in the onset of puberty. In this study, we aimed to investigate the role of central stimulants in the onset of PT by measuring plasma kisspeptin levels in patients with PT. 

## METHODS

The patient group included 20 girls aged between 3-7 years who presented to the outpatient clinic of our pediatric endocrinology unit with a complaint of unusual breast development. The control group consisted of 20 age-matched healthy girls who were admitted to the hospital for routine health control and who were not on any medication. The study was approved by the institutional ethics review board and written informed consent was obtained from all the families. Information was obtained on age at onset of PT, progression rate of the symptoms , annual growth rate, medications used, and maternal menarche age. Height standard deviation score (HSDS) and body mass index SDS (BMISDS) were calculated in the patients and controls according to Turkish reference values (18). Bone age (BA) of the patients and controls were assessed by the Tanner-Whitehouse method. Breast development was evaluated according to the Tanner staging (19). Uterine and ovarian sonographic (USG) examinations were performed and interpreted by the same investigator using a USG apparatus (Acuson Antares, Siemens 2010) with a 1-4 mHz transducer. The USG scans were performed transabdominally when the patients/controls had a full bladder, obtained by voluntary urine retention and oral administration of fluids. On pelvic USG, ovarian and uterine volumes [longitudinal diameter (A), largest transverse diameter (B), largest anterior-posterior diameter (C)] were calculated using the ellipsoid formula (AxBxCx0.52). Endometrial thickness and presence/absence of follicular cysts were also investigated. On USG evaluation, a uterine volume of < 3cm3, an ovarian volume of <1 cm3 and an endometrial thickness of <1mm were considered as prepubertal (20,21,22). In the patient and control groups, basal free thyroxine (fT4), thyroid-stimulating hormone (TSH), LH, FSH, SHBG, E2 and PRL levels were measured by immunochemiluminometric assay (ICMA) on an Immulite 2000 analyzer (Siemens; catalog numbers: FSH:L2KFS2, LH:L2KLH2, E2:L2KE22, PRL:L2KPR2, SHBG:L2KSH2). GnRH stimulation test was performed in all patients with PT.

Subjects with basal LH values of <0.3 mIU/mL (ICMA), peak LH levels of <5 mIU/mL during the GnRH test, with BA/chronological age ratios of <1, a normal annual height increase according to age and a regression or a cessation of breast development at the end of 1-year monitoring, and who showed no other signs of puberty such as axillary or pubic hair development were considered as having PT (23,24,25,26,27,28). These patients were evaluated by measurement of FSH, LH, E2, and PRL levels and by pelvic USG every 3 months for a period of 1.5 years. During this follow-up period, no significant changes were observed in the hormonal levels or pelvic USG findings, and none of the patients developed other signs of precocious puberty.

**Measurement of kisspeptin:** For measurement of kisspeptin level, blood samples were drawn from the patient and control groups into tubes containing EDTA and aprotinine, a protease inhibitor. The tubes were immediately centrifuged at 1.600 rpm at a temperature of 4°C for 15 minutes, and the samples were stored at -80°C until time of measurement. Before initiating the measurement, the peptides were extracted from the plasma. For measurements, the following steps were followed using the buffers and columns required along with the kit: The first step was to acidify the plasma using an equal amount of buffer A (Catalogue No: RKBA-1). After being well-mixed, the sample was centrifuged at 6 000-17 000 rpm at a temperature of 4°C for 20 minutes. Then, the SEP-COLUMN that contains 200 mg C18 (Catalogue no: RKSEPCOL-1) was equilibrated by washing with 1 mL buffer B (Catalogue no: RKBB-1) once. Then, the sample was washed twice with 3 mL buffer A and the resulting solution was separated. The peptides were then eluted with 3 mL buffer B and the eluents were collected into polystyrene tubes.

Kisspeptin was measured using a commercial enzyme immunoassay (EIA) kit called Kiss-1 [112-121] Amide/ Kisspeptin-10 / Metastin (45-54) Amide (Human) (Cat# EK-048-56), (Phoenix Pharmaceuticals, Inc., Burlingame, California, USA) and according to the directions provided in the kit content. At the end of the study, ELISA plate (TRITURUS, Barcelona, Spain) was read at 450 nm, and the results were calculated according to standards (ng/mL).

**Statistical Methods:** Statistical analyses were performed using SPSS for Windows, version 11.0. All the data were reported as means±SD. Normality for continued variables in the groups was determined by Shapiro-Wilk test. The variables showed normal distribution (p>0.05), so the unpaired t-test was used for comparisons of the groups for variables and the Pearson's correlation test - for correlations between the variables. A p-value of <0.05 was considered significant.

## RESULTS

Clinical and ultrasound findings of 20 girls diagnosed with PT and of 20 healthy girls are given in Table 1. No statistically significant difference was found between patient and control groups in terms of auxological characteristics (age, HSDS, BMISDS, BA) (p>0.05).In the PT group, all patients were evaluated as Tanner stage-2 breast development, except for one girl who showed Tanner stage-3 breast development. At presentation, breast development was bilateral in 60% and unilateral in 40% of the patient group. Of the patients with unilateral breast development, 62.5% had left and 37.5% had right breast development. No statistically significant differences were found between the patient and control groups in terms of ovarian and uterine volumes (p>0.05) (Table 1). The basal E2, PRL, SHBG, LH, FSH and kisspeptin levels in both PT patients and controls as well as the peak FSH and LH response values to GnRH stimulation in the patient group are presented in Table 2. While the patient and control groups did not statistically significantly differ in terms of basal FSH, LH, SHBG, and E2 levels (p>0.05), basal PRL levels were higher in the patient group (p<0.05) (Table 2). The mean peak values of LH and FSH during GnRH test in the patient group are shown in Table 2.

Higher FSH peak values after GnRH stimulation were consistent with the diagnosis of PT. Peak LH levels of the patients were within the prepubertal range. Kisspeptin levels were significantly higher in the patient group than in the control group ( p<0.05) (Table 2).In the patient group, a statistically significant positive correlation was found between kisspeptin level and basal E2, LH, FSH, PRL levels, and GnRH-stimulated LH peak values (r=0.45, p<0.05; r=0.49, p<0.05; r=0.42, p<0.05; r=0.45, p<0.05; and r=0.49, p<0.05, respectively). There was no significant correlation between kisspeptin level and E2, FSH, LH and PRL levels in the control group. We did not find any correlations between kisspeptin levels and BMISDS of patients and controls. All the cases with PT were followed up for a mean period of 15.2±1.57 months. Breast findings resolved within 6±1.45 months in 3 cases with unilateral breast enlargement; the others did not show any progression. The breast findings resolved in five cases with bilateral breast enlargement within 10±1.62 months; in the others, breast growth regressed but did not disappear. When the patients with PT were divided with respect to the resolution of breast tissue, there were no significant differences between kisspeptin levels of both groups. In the PT group, no signs of early puberty were seen at follow-up.

## DISCUSSION

In our study, anthropometric measurements of the PT group were within normal limits for age, and the mean values ofanthropometric values in the PT group were not different from those in the control group. Moreover, in the PT patients, at the and of a follow-up of nearly 1.5 years, no findings suggesting early puberty were observed based on physical examination and pelvic USG evaluation. These findings confirmed a diagnosis of PT in the patient group.

The etiology of PT remains poorly understood. Various hypotheses have been proposed, and PT most likely arises from any one or a combination of these mechanisms. Hypothyroidism typically causes pubertal delay but may also induce a syndrome of pseudoprecocity manifested as breast enlargement and vaginal bleeding in girls (29). In our patients, fT4 and TSH levels were within normal limits. Oral ingestion of estrogen-containing foods or medications can cause PT (7).

E2 levels in girls slowly rise from infancy through childhood until puberty, but some girls with PT may have an increased sensitivity of breast tissue to small amounts of estrogens. Some girls may have a prolonged minipuberty with delayed inactivation of the HPG axis, leading to increased estradiol levels and, consequently, to PT (30,31). Increased peripheral aromatase activity may be another source of estrogens. Obese or overweight girls may have increased peripheral aromatase activity and breast development (8). In our study, BMI values were within normal limits and were comparable in the patient and control groups. Basal gonadotropin levels are considered a leading sign in deciding whether isolated breast development is accompanied by the activation of HPG axis, in other words, whether “central puberty” has begun (32). Some studies have reported LH secretion during sleep in children with PT to be similar to that seen in puberty (33), whereas others have not confirmed these findings (34). In prepubertal girls who show the initial symptoms of puberty ( breast enlargement), LH values of >0.3 mIU/mL (by ICMA) and LH peak values with GnRH stimulation of >5mIU/mL (by ICMA) indicate central precocious puberty (24,25). In our study, the basal and peak LH values in our patients were <0.3 mIU/mL and <5 mIU/mL, respectively, and these values were not different from those in the control group. PT is typically associated with increased FSH and increased inhibin B secretion. Breast enlargement may be a consequence of fluctuations in the female childhood HPG axis activation, with temporary FSH-stimulated increases in ovarian steroids (5,10). In our study, FSH predominance was observed in GnRH stimulation test.Although the mechanisms used to control or initiate episodic release of hypothalamic GnRH remain incompletely understood, some neuropeptides like GABA, dopamine and serotonin are known to play an inhibitory role, while others, such as kisspeptin, noradrenaline and glutamate, stimulate the gonadotropin release (35,36). Recently discovered kisspeptin is a neuropeptide encoded by Kiss-1 gene (1q32). Kisspeptin, which is synthesized in the central nervous system, stimulates gonadotropin release by binding to GPR54, located on GnRH neurons in the hypothalamus (12,13,14,15). This protein, GPR54, appears to be necessary in mammals for the pulsatile FSH and LH release at the onset of puberty. In men, it has been shown that inactivating mutation in GPR54 resulted in decreased GnRH secretion and hypogonadism (37). In patients with GPR54 mutations, peak LH and FSH levels were found to be decreased, while these values were increasing with GnRH stimulation. These findings support the view that kisspeptin/GPR54 signal system is important in pubertal timing. Otherwise, in some girls, an increased expression of the Kiss-1 gene leading to increased kisspeptin levels may be the cause of central precocious puberty, or could be the result of increased activation of the GPR54 signal system. Recent studies show that kisspeptin plays an important role in the onset of puberty (9) because the girls diagnosed with precocious puberty had higher plasma levels of kisspeptin in earlier studies (38). Animal studies have shown that peripheral or cerebroventricular kisspeptin injections increase the peak values of LH and FSH (39,40). In a recently performed study, kisspeptin-54 was given to male volunteers and an increase in their LH, FSH and testosterone levels was observed (13). These findings show that kisspeptin/GPR54 signal system plays a crucial role in triggering FH and FSH secretions. In PT patients, FSH predominance suggests the role of central stimulants in the occurrence of PT. In this research also, PRL levels were higher in PT patients than in the control group, and they were significantly correlated with kisspeptin levels. Experimental studies have shown that intraventricular injection of kisspeptin-10 inhibits the dopaminergic neurons and increases PRL levels (41,42). In our study, the significantly higher plasma levels of kisspeptin and PRL as well as the significant positive correlation between plasma levels of kisspeptin and basal hormone levels (FSH, LH, E2, PRL) in the girls with PT suggest that PT may be directly related to a premature increase of kisspeptin leading to premature activation of the HPG axis or that this relationship may be an indirect one, via a kisspeptin-induced increase in PRL. Studies on kisspeptin levels in childhood are limited. Further studies are warranted to achieve a better understanding about the role of kisspeptin in the occurrence of PT or to document the usefulness of kisspeptin as a marker in the diagnosis of PT.

## Figures and Tables

**Table 1:**
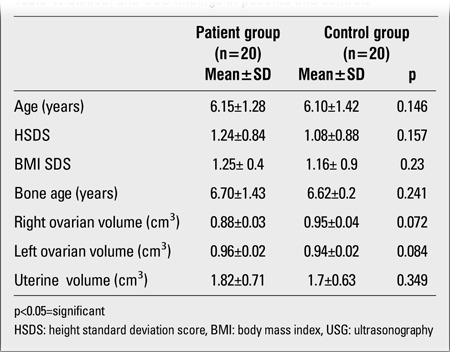
Clinical and USG findings in patients and controls

**Table 2:**
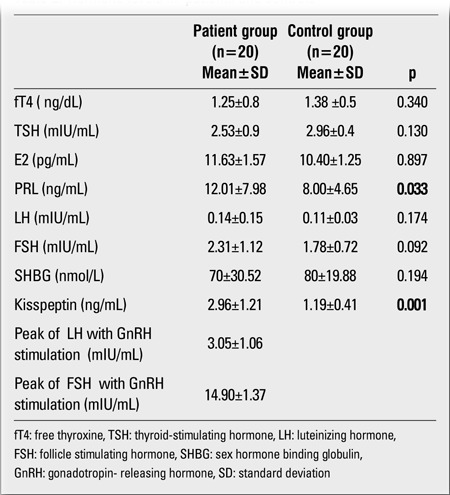
Hormone levels in patients and controls
